# Multiple maternal occupational exposures during pregnancy and newborn size at birth: analysis of the ELFE cohort, a French longitudinal study of children

**DOI:** 10.5271/sjweh.4289

**Published:** 2026-09-01

**Authors:** Marie Tartaglia, Kellian Jaunas, Sabyne Audignon-Durand, Marie-Tülin Houot, Maxime Turuban, Nel Roeleveld, Jack Siemiatycki, Marie Noëlle Dufourg, Camille Carles, Corinne Pilorget, Sanni Uuksulainen, Michelle C Turner, Alexis Descatha, Ronan Garlantézec, Fleur Delva

**Affiliations:** 1 University of Bordeaux, INSERM, Bordeaux Population Health Center, Epicene Team, U1219, Bordeaux, France.; 2 Consultation de pathologie professionnelle et environnementale, service santé travail environnement, CHU de Bordeaux, France.; 3 Direction Appui, Traitements et Analyse des données, Unité AMETIS, Santé Publique France.; 4 Barcelona Institute for Global Health (ISGlobal), Barcelona, Spain.; 5 Radboud university medical center, Radboud Institute for Health Science, Nimègue, The Netherlands.; 6 CRCHUM, University of Montreal, Montreal, Canada.; 7 Unité Mixte de Recherche ELFE, Ined-Inserm-EFS, France.; 8 Direction santé environnement travail, Santé Publique France, France.; 9 Finnish Institute of Occupational Health (FIOH), Helsinki, Finland.; 10 Universitat Pompeu Fabra (UPF), Barcelona, Spain.; 11 Spanish Consortium for Research and Public Health (CIBERESP), Instituto de Salud Carlos III, Madrid, Spain.; 12 Univ Angers, CHU Angers, Univ Rennes, Inserm, EHESP, Irset (Institut de recherche en santé, environnement et travail), UMR_S 1085, Ester team, SFR ICAT, CAPTV/Prevention, Angers, France.; 13 Indiana University School of Public Health-Bloomington (IU SPH-B), IN, USA / Department of Occupational Medicine, Epidemiology and Prevention, Donald and Barbara Zucker School of Medicine, Hofstra/Northwell, USA.; 14 CHU Rennes, University of Rennes, Inserm, EHESP, IRSET (Institut de Recherche en Santé, Environnement et Travail) - UMR_S 1085, Rennes, France.

**Keywords:** fetal growth, hypothesis-driven, pregnant worker

## Abstract

**Objective:**

We aimed to study multiple occupational exposures selected using an priori hypothesis as well as small-for-gestational age (SGA), birthweight (BW), and head circumference (HC).

**Methods:**

We analyzed data from the Étude Longitudinale Française depuis l'Enfance (ELFE) cohort (N=12 851 mother-child pairs with a gestational age >33 weeks). The outcomes of interest were SGA, BW, and HC. Of 47 factors (17 chemical, 8 physical, 4 biological, 12 biomechanical, 3 organizational and 3 psychosocial) to which mothers were exposed in their occupation assessed by job-exposure matrices in this cohort, we included those for which the epidemiological or experimental literature suggests an association with fetal growth. Logistic and linear regression models that included all preselected exposure variables were performed and adjusted for potential confounders. We conducted additional analysis stratified by trimester of pregnancy.

**Results:**

The logistic model, which included all preselected variables, reported that – for all women – exposure to ultrafine particles (UFP) increased SGA risk [odds ratio (OR) 1.46, 95% confidence interval (CI) 1.12–1.90] while standing decreased it (OR 0.82, 95% CI 0.67–1.00). Among mothers who stopped working during the first/second trimester of pregnancy, we found the same SGA risk for UFP; exposure to high strain also increased the risk (OR 1.81, 95% CI 1.18–2.83). Among those who stopped working during the third trimester, exposure to oxygenated solvents increased SGA risk (OR 2.24, 95% CI 1.08–4.41). In the linear model, for all women, there was a decrease in BW (β -34 grams, 95% CI -66– -1.1) among those exposed to UFP. For mothers who stopped working during the first/second trimester, exposure to vibration increased BW, while among those who stopped working during the third trimester, exposure to night work increased BW. Exposure to oxygenated solvents decreased HC (β -0.22 cm, 95% CI -0.44–0.00) among all women.

**Conclusion:**

These findings suggest the possible influence of chemical and strenuous factors at work on fetal growth, particularly depending on the trimester in which pregnant mothers stopped working. While the combined assessment of multiple exposures did not reveal clear associations, further studies are needed to explore potential interactions and underlying biological mechanisms.

The vast majority of women work during pregnancy (1) – 80.2% of women in 13 European birth cohorts – and may face different occupational exposures to chemical, physical, biological, strenuous, organizational, and psychosocial factors. This highlights the relevance of understanding the potential effects of maternal occupational exposures on newborn size at birth [small-for-gestational age (SGA), birthweight (BW) and head circumference (HC)].

In particular, newborn size at birth is highly sensitive to environmental influences, and exposure to certain occupational factors during pregnancy has been associated with adverse outcomes. The epidemiological literature demonstrates how factors affect newborn size at birth outcomes, including several chemicals such as organic solvents (2, 3), pesticides (4–6), iron (7), nickel (8), ultrafine particles (UFP) (9, 10), welding fumes (7), and polycyclic aromatic hydrocarbons (PAH) (11–13). Strenuous factors also have an effect, including physical effort (14–20), carrying loads (21), load handling (22), standing (21, 23–26), vibrations (27), and night work (28). Additional psychosocial factors such as decision-making (29–31), demands (30), and strain (14, 15, 30–32) are known to impact BW. The effect of solvents (2), UFP (10) for chemicals, and a long-period of standing (21) can result in smaller HC.

Experimental studies have supported some of these associations (33–39). Animal and in vitro models have shown that prenatal exposure to several chemicals can induce oxidative stress, inflammation, endocrine disruption, and impaired placental development, affecting fetal growth. Experimental evidence also suggests that particulate matter may alter placental vascularization and nutrient transport.

The epidemiological studies cited above have focused mainly on individual occupational exposures, without considering them in combination. A recent study showed that biomechanical factors were the most frequent workplace exposures, over 40% of pregnant women are exposed to ≥3 potential occupational risk factors for pregnancy, and about 30% are exposed to ≥5 (40). More recently we studied the occupational exposome of women during pregnancy by assessing 47 factors during pregnancy; we revealed that women were exposed to a median of 6 occupational factors (41), thus highlighting the reality of occupational situations and the need to account for multiple exposures. In addition, we described different profiles of co-exposure that may be of interest in relation to the fetal growth restriction. Data-driven approaches can also be useful in studying maternal occupational exposures and newborn size at birth, as they can handle large datasets and generate new hypotheses (42). However, they have shown certain limitations when compared in the literature, such as inconsistencies in the selection of variables and providing no further understanding of the biological and pathophysiological mechanisms that may be involved (43). In a previous study, we used this approach to select occupational exposures with the most significant effects on newborn size at birth that reveal unexpected findings (44). Some exposures did not emerge as significant, even though it is known from the literature that they have an effect on fetal growth. Conversely, some exposures were identified as significant, even though, to date, no data from the epidemiological and experimental literature has found an effect on fetal growth. In light of these limitations, approaches based on a priori hypotheses offer a complementary alternative (43) as they structure their analysis around plausible and well documented associations.

We therefore aimed to study multiple occupational exposures, selected using a hypothesis based on a literature review, and birth outcomes characterized by BW, SGA, and HC.

## Methods

### Population study

The Étude Longitudinale Française depuis l'Enfance (ELFE study) is the first French national study dedicated to monitoring children’s health from birth to adulthood. The ELFE cohort methodology has previously been described in detail (45). In brief, ELFE employed a stratified sampling approach, taking into consideration the size of each maternity ward, to facilitate oversampling in large maternity units. Among the 544 maternity departments identified in France in 2011, 349 were selected and 320 agreed to participate. Recruitment then took place in four periods within each maternity department that agreed to participate. Single or multiple live births ≥33 weeks of gestation by mothers whose age was ≥18 years, who were not intending to leave France within three years, and who had signed an informed consent, were included in the study. Fifty-nine families requested the destruction of their data, meaning that 18 270 children were included in the ELFE cohort follow-up.

For this sample, we excluded multiples pregnancies (N=574), major congenital malformations (N=377) (as defined by the European Platform on Rare Disease Registration, the European agency that monitors congenital anomalies), and children of mothers who did not work during their pregnancies (N=4137).

The Consultative Committee for the Treatment of Information in Health Research (CCTIRS), the National Commission for Data Protection and Liberties (Cnil), and the Committee for the Protection of Persons (CPP). approved ELFE The National Council for Statistical Information (CNIS) also endorsed the study.

### Data collection

Socio-demographic data were collected at the maternity facility through face-to-face interviews with mothers, while medical data were collected from medical records. Information on women’s occupations during pregnancy, including job title, was collected in the face-to-face interviews at during the maternity stay and completed using a questionnaire conducted two months later. More details are available at: www.elfe-france.fr/en.

### Outcomes

BW refers to the weight of the newborn at birth, expressed in grams. SGA refers to bottom 10^th^ percentile for sex, gestational age, maternal height and weight, and parity, using the Epopé curves (46). HC is the fronto-occipital head circumference measured at birth, expressed in centimeters.

### Assessment of occupational exposure

Participants' self-reported jobs were coded by one expert according to the International Standard Classification of Occupations (ISCO-1968) and the French classification of professions and socio-professional categories (PCS-2003) for the occupations. Industry activities were coded according to the International Standard Industrial Classification of All Economic Activities (ISIC-1975) and the French nomenclature of activities (NAF-2003). All mothers included in this study held only one job during their pregnancy.

Based on this information, we applied several job-exposure matrices (JEM) to assess occupational exposure to 47 factors (17 chemical, 8 physical, 4 biological, 12 strenuous, 3 organizational, 3 psychosocial factors) (supplementary material, www.sjweh.fi/article/4289, appendix 1). We selected general population JEM, preferably from the French (or European or North American) population, and characterizing the exposure close to 2011. The description and the profile of co-exposure to the occupational exposures have been described in detail in a previous publication (41).

### Selection of occupational exposures associated with SGA, BW, and HC

From the 47 occupational exposures assessed in our study, we selected the exposure for which ≥2 epidemiological studies have found a statistically significant association with fetal growth or for which there is a strong hypothesis in experimental studies (table 1, supplementary appendix 2). The strong hypothesis in experimental studies was defined through a consensus among three of the co-authors (Marie Tartaglia, Fleur Delva, Ronan Garlantézec). Drawing on the findings of the epidemiological literature, we also considered the existence of interaction between exposures in relation with the outcomes when previous studies reported significant interactions.

**Table 1:** Summary of the occupational exposure selection concerning intrauterine growth outcomes. Details are presented in supplementary appendix 2. [SGA=small-for-gestational age; HC=head circumference.]

Occupational exposures	SGA and BW				HC		
	Epidemiological literature with ≥2 studies finding significant association	Experimental study with strong hypothesis	Included in the study		Epidemiological literature with ≥2 studies finding significant association	Experimental study with strong hypothesis	Included in the study
Chlorinated solvents	Yes	Yes	Yes		Yes/No	Yes	Yes
Oxygenated solvents	Yes	Yes	Yes		Yes/No	Yes	Yes
Petroleum solvents	Yes	Yes	Yes		No	No	No
Detergents	No	No	No		No	No	No
Pesticides	Yes	Yes	Yes		No	Yes	Yes
Arsenic	No	No	No		No	No	No
Cadmium	No	No	No		No	No	No
Chromium	No	No	No		No	No	No
Iron	No	Yes	Yes		No	No	No
Lead	No	No	No		No	No	No
Nickel	No	No	No		No	No	No
UFP	Yes	Yes	Yes		No	Yes	Yes
Welding fumes	No	Yes	Yes		No	No	No
Polycyclic Aromatic Hydrocarbon	Yes	Yes	Yes		No	No	No
Benzo-a-pyrene	No	No	No		No	No	No
Carbon monoxide	No	Yes	Yes		No	No	No
Endocrine disruptor	No	No	No		No	No	No
Ionizing radiations	No	No	No		No	No	No
Low-frequency magnetic fields	No	No	No		No	No	No
Non-thermal intermediate frequency	No	No	No		No	No	No
Radiofrequency (E and H field)	No	No	No		No	No	No
Thermal intermediate frequency (E and H field)	No	No	No		No	No	No
Ultraviolet	No	No	No		No	No	No
Airborne germs	No	No	No		No	No	No
CMV, parvovirus B19	No	No	No		No	No	No
Hepatitis A, E	No	No	No		No	No	No
Hepatitis B, C, HIV	No	No	No		No	No	No
Intensity of physical effort	Yes	Yes	Yes		No	No	No
Carrying loads 10-25 kg	No	Yes	Yes		No	No	No
Handling of loads >4 kg	No	Yes	Yes		No	No	No
Arms in the air above the shoulders	No	No	No		No	No	No
Standing	Yes	Yes	Yes		No	Yes	Yes
Kneeling or squatting	No	No	No		No	No	No
Leaning forward or sideways	No	No	No		No	No	No
Repetitive actions	No	No	No		No	No	No
Using vibrating tools	No	Yes	Yes		No	No	No
Driving construction machinery	No	No	No		No	No	No
Driving a vehicle	No	No	No		No	No	No
Using a computer screen or control panel	No	No	No		No	No	No
Night work	No	Yes	Yes		No	No	No
Interruption of tasks	No	No	No		No	No	No
Working outdoors	No	No	No		No	No	No
Low decision authority	No	No	No		No	No	No
High job demands	No	Yes	Yes		No	No	No
High strain	Yes	Yes	Yes		No	No	No

For each selected exposure, we built a categorical variable based on the probability for which an association has already been found in the literature. When multiple studies found an association, the most specific threshold was chosen. When the percentage of exposure was low, we only considered exposed whatever the probability of exposure.

For the statistical analyses, we finally selected 17 exposures for SGA and BW (chlorinated, oxygenated and petroleum solvents, pesticides, iron, UFP, welding fumes, polycyclic aromatic hydrocarbon, carbon monoxide, intensity of physical effort, carrying loads, handling of loads, standing, using vibrating tools, night work, high job demands, and high strain), and the interaction between carrying loads and high strain (29) for SGA; and five for HC (chlorinated and oxygenated solvents, pesticides, UFP, and standing).

### Statistical analysis

The study of correlations between occupational exposures revealed a correlation >0.8 between iron and welding fumes, which led us to exclude iron from our analyses. Welding fumes and HAP were also excluded from our analyses on SGA because of the smaller number of women exposed with SGA (10 and 3 women, respectively).

We performed logistic regression model for SGA and linear regression models for BW and HC, including all preselected exposure variables. In these models, the “not exposed” of each exposure variable group was the reference. We performed analyses that also considered the trimester of pregnancy in which mothers stopped working. We also presented results of an exposome-wide association study (EWAS), for each exposure selected and the three outcomes. EWAS corresponds to the realization of single-exposure approaches based on logistic (for SGA) and linear (for BW and HC) regressions for all the exposure of interest separately.

Missing data on occupational exposures were imputed using a method based on chain equations with the mice package in R software to generate five tables, by five iterations. The imputation model contains all the occupational exposure variables, and all covariates.

Based on a directed acyclic graph (DAG) (supplementary appendix 3), all models were adjusted for educational level (low, high school, university), prepregnancy body mass index (BMI) (<18.5, 18.5–24.9, 25–29.9 and ≥30 kg/m^2^), tobacco use (yes, no), alcohol consumption (continuous, number of glasses per day) during pregnancy, and gestational age (continuous) for analysis on SGA. For analysis on BW and HC, we additionally adjusted for infant sex (female, male). This variable was not included in the SGA analysis since this factor is already considered in the construction of this outcome variable.

Effect estimates were presented as odds ratios (OR) for logistic regressions; and beta (β) for linear regressions.

The analyses were performed using R Core Team (2023) software, using the mice package (47) for multiple imputation and epiDisplay for logistic regression.

## Results

### Population

Of all the women who participated in the ELFE cohort, 12 851 constituted our study population (supplementary appendix 4). The mean maternal age at inclusion was 31.1 [standard deviation (SD) 4.7] years. During pregnancy, <20% of mothers were smokers (N=2279; 17.9%) or consumed alcohol (N=2077; 18.7%). The average duration of work during pregnancy was 27 (SD 7.9) weeks, with 57.3% (N=7179) of mothers continuing to work into the third trimester. A total of 15.1% (N=1663) of women had access to or benefitted from specific adjustments in the workplace. The mean gestational age at birth was 39.3 (SD 1.4) weeks. The mean BW was 3338.8 (SD 471.3) grams, and 8.6% of babies had a SGA. The mean HC was 34.4 (SD 1.4) cm (table 2).

**Table 2:** Characteristics of the population. Elfe study, France, N=12 851. [SGA=small-for-gestational age; SD=standard deviation].

		All N=12 851			SGA N=1085			No SGA N=11 266	
		N (%) ^a^	Mean (SD)		N (%) ^a^	Mean (SD)		N (%) ^a^	Mean (SD)
**Socio economic**									
Maternal age (years)			31.1 (4.7)			31.4 (4.9)			31.0 (4.7)
	Unknown	52			0			6	
Mother’s nationality									
	French	12 161 (95.5)			1030 (95.3)			10 717 (95.5)	
	Foreign	571 (4.5)			51 (4.7)			497 (4.4)	
	Stateless	4 (0.0)			0 (0.0)			4 (0.0)	
	Unknown	115			4			48	
Maternal education level ^b^									
	Low	249 (1.9)			32 (2.9)			212 (1.9)	
	High school	3692 (28.8)			354 (32.6)			3192 (28.3)	
	University	8863 (69.2)			699 (64.4)			7861 (69.8)	
	Unknown	47			0			1	
In a relationship		12 287 (96.5)			1026 (95.1)			10 837 (96.7)	
	Unknown	120			6			55	
Marital status									
	Celibate	4474 (35.6)			439 (41.2)			3887 (35.1)	
	Partner	2221 (17.7)			187 (17.6)			1976 (17.8)	
	Married	5733 (45.7)			429 (40.3)			5091 (46.0)	
	Divorced	116 (0.9)			9 (0.8)			106 (1.0)	
	Widow	13 (0.1)			1 (0.1)			11 (0.1)	
	Unknown	294			20			195	
Monthly household income (euros)									
	<2500	2289 (18.9)			223 (21.9)			1975 (18.6)	
	2500–4000	6713 (55.5)			578 (56.8)			5914 (55.8)	
	>4000	3095 (25.6)			217 (21.3)			2718 (25.6)	
	Unknown	754						659	
**Before pregnancy**									
Body mass index (kg/m^2^)									
	<18.5	932 (7.4)			85 (7.8)			819 (7.3)	
	18.5–25.0	8573 (67.6)			686 (63.2)			7669 (68.1)	
	25.0–30.0	2116 (16.7)			199 (18.3)			1870 (16.6)	
	≥30	1052 (8.3)			115 (10.6)			908 (8.1)	
Diabetes (type 1 or 2)		115 (0.9)			12 (1.1)			98 (0.9)	
	Unknown	371			15			236	
Chronic high blood pressure		284 (2.3)			45 (4.2)			226 (2.0)	
	Unknown	268			12			137	
**During pregnancy**									
Parity									
	Primiparous	6153 (48.3)			552 (51.2)			5389 (48.1)	
	Multiparous	6587 (51.7)			527 (48.8)			5826 (51.9)	
	Unknown	111			6			51	
Number of deliveries			1.4 (0.7)			1.4 (0.8)			1.4 (0.7)
	Unknown	6857			598			5926	
Hypertensive disorders		405 (3.2)			72 (6.7)			321 (2.9)	
	Unknown	360			17			198	
Preeclampsia		149 (1.2)			32 (3.0)			111 (1.0)	
	Unknown	360			17			198	
Smoking		2279 (17.9)			306 (28.3)			1899 (17.0)	
	Unknown	136			3			73	
Alcohol consumption									
	No	9024 (81.3)			757 (82.0)			7981 (81.4)	
	<1 glass per month	1637 (14.7)			122 (13.2)			1445 (14.7)	
	≥1 glass per month	440 (4.0)			44 (4.8)			377 (3.8)	
	Unknown	1750			162			1463	
Stopping the professional activity									
	1^st^ trimester	1283 (10.2)			118 (11.0)			1121 (10.1)	
	2^nd^ trimester	4074 (32.5)			367 (34.2)			3607 (32.5)	
	3^rd^ trimester	7179 (57.3)			588 (54.8)			6387 (57.5)	
	Unknown	315			12			151	
Duration of work (weeks)			27.4 (7.9)			27.1 (8.1)			27.4 (7.9)
	Unknown	376			17			216	
Hospitalization		1857 (14.6)			189 (17.5)			1597 (14.2)	
	Unknown	130			6			55	
Workplace adjustment		1663 (15.1)			130 (14.0)			1480 (15.3)	
	Unknown	1828			157			1589	
**Childbirth**									
Gestational age (weeks of gestation)			39.3 (1.4)			39.2 (1.6)			39.3 (1.4)
	Unknown	200			2			19	
Mode of delivery									
	Caesarean	2141 (17.1)			245 (22.9)			1826 (16.5)	
	Vaginal delivery	10 376 (82.9)			824 (77.1)			9257 (83.5)	
	Unknown	334			16			183	
Infant’s sex									
	Female	6283 (49.3)			552 (50.9)			5497 (48.8)	
	Male	6458 (50.7)			532 (49.1)			5761 (51.2)	
	Unknown	110			1			8	
Birthweight (in grams)			3338.8 (471.3)			2691.4 (377.6)			3401.6 (427.5)
	Unknown	272			2			21	
Head circumference (in centimetres)			34.4 (1.4)			33.1 (1.3)			34.6 (1.4)
	Unknown	1439			124			978	

^a^ Percentage were calculated excluding the category “Unknown” ^b^ Low was defined as never attended school, primary and middle school; High school defined as certificate of vocational aptitude, professional qualifications, high school; University defined as higher education.

### Occupational characteristics during pregnancy

The occupational groups of the mothers during pregnancy were predominantly professional and technical (41.2%), followed by clerical (23.9%), service (16.6%), sales (11.1%), managerial (3.6%), production (3.1%), and agricultural (0.8%) (supplementary appendix 5).

The chemical factors to which mothers were most frequently exposed during pregnancy were carbon monoxide (10.0%), petroleum solvents (6.6%), and UFP (5.8%). With regard to strenuous factors, 37.4% of women were exposed to standing. More than half were exposed to high strain (51.8%), and 40.0% were exposed to high job demands (table 3). The correlations between these occupational exposures are presented in supplementary appendix 6.

**Table 3:** Description of occupational exposure of women during pregnancy. Elfe study, France, N=12 851.

		Not exposed	Uncertain exposed		Exposed	Missing
			Likely exposed	Certainly exposed		
		N (%)	N (%)	N (%)	N (%)	
**Chemical factors**						
Solvents						
	Chlorinated	12 275 (98.5)	-	-	188 (1.5)	388
	Oxygenated	9592 (77.5)	809 (6.5)	1731 (14.0)	243 (2.0)	476
	Petroleum	11 573 (93.4)	-	-	815 (6.6)	463
Pesticides		10 078 (97.0)	-	-	312 (3.0)	2461
UFP		9527 (80.6)	1610 (13.6)		689 (5.8)	1025
Welding fumes		12 456 (98.6)	-	-	182 (1.4)	213
Polycyclic aromatic hydrocarbons		12 612 (99.8)	-	-	25 (0.2)	214
Carbon monoxide		11 039 (90.0)	-	-	1234 (10.0)	578
**Strenuous factors**						
Intensity of physical effort		1626 (13.5)	4690 (39.0)	5013 (41.7)	703 (5.8)	819
Carrying loads 10–25 kg		10 004 (82.7)	1200 (9.9)	736 (6.1)	161 (1.3)	750
Handling of loads >4 kg		6964 (57.9)	1491 (12.4)	1686 (14.0)	1895 (15.7)	815
Standing		2632 (21.9)	2364 (19.6)	2541 (21.1)	4496 (37.4)	818
Using vibrating tools		11 945 (95.7)	427 (3.5)		105 (0.8)	374
**Organizational factors**						
Night work		10 803 (92.5)	-	-	876 (7.5)	1172
**Psychosocial factors**						
High job demands		2555 (22.0)	4407 (38.0)		4642 (40.0)	1247
High strain		2504 (21.6)	3092 (26.6)		6008 (51.8)	1247

The description of population and occupations according to occupational exposures is presented in supplementary appendix 7.

### Multiple occupational exposures during pregnancy and their effects on intrauterine growth

*SGA.* Results of EWAS for SGA are presented in supplementary appendix 8, table S1. Briefly, it revealed that exposure to UFP was significantly associated with SGA.

**Figure 1:**
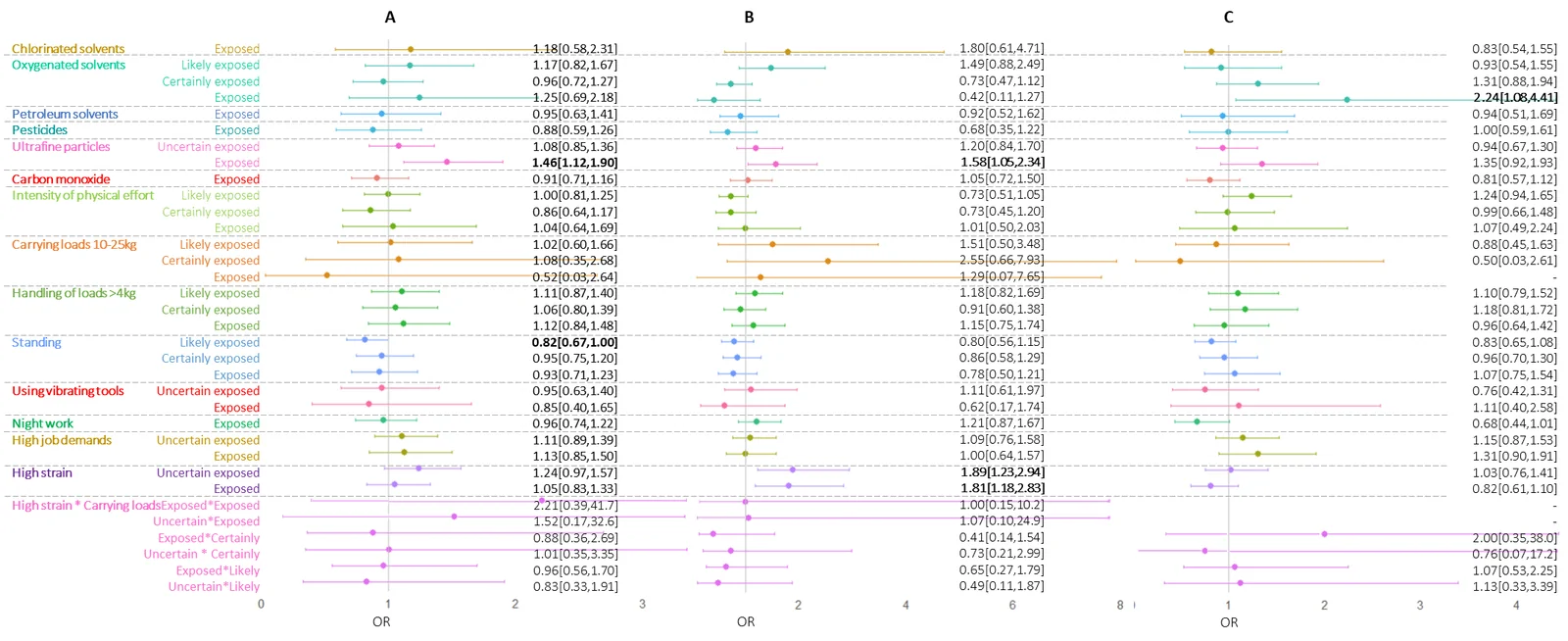
Relationship between occupational exposures and SGA, for all women (A), women who stopped working during the first/second trimester of pregnancy (B), or the third trimester of pregnancy (C). Results of logistic regression model adjusted for education level, tobacco, alcohol and BMI, with modality “not exposed” as the reference. Elfe study, France, N=12 351.

We observed an increased risk of SGA (OR 1.46 95% CI 1.12–1.90) with exposure to UFP, and a decreased risk of SGA (OR 0.82 95% CI 0.67–1.00) with likely exposure to standing, among all women (figure 1A).

Among women who stopped working during the first/second trimester of pregnancy, we observed an increased risk of SGA with exposure to UFP, and high strain (OR 1.58 95% CI 1.05–2.35 and OR 1.81 95% CI 1.18–2.83, respectively).

Among those who stopped working during the third trimester of pregnancy, exposure to UFP, and oxygenated solvents increased the risk of SGA (OR 1.35 95% CI 0.92–1.93 and OR 2.24 95% CI 1.08–4.41, respectively), and exposure to night work decreased the risk of SGA (OR 0.68 95% CI 0.44–1.01).

We found that 23.1% of the women exposed to UFP were other service workers and 22.8% were technical saleswomen or commercial travelers. Of the women exposed to oxygenated solvents, 48.1% were chemists, biologists, and related workers and 32.1% were hairdressers, beauticians. Of those exposed to high strain, 11.9% were clerical and related workers, 11.8% were cleaners and helpers, 11.5% were bookkeepers, cashiers, and 10.6% were saleswomen, shop assistants (supplementary appendix 7).

*BW.* Results of EWAS for BW are presented in supplementary appendix 8, table S2. Briefly, it revealed that exposure to chlorinated solvents and certainly exposure to intensity of physical effort were significantly associated with BW.

**Figure 2:**
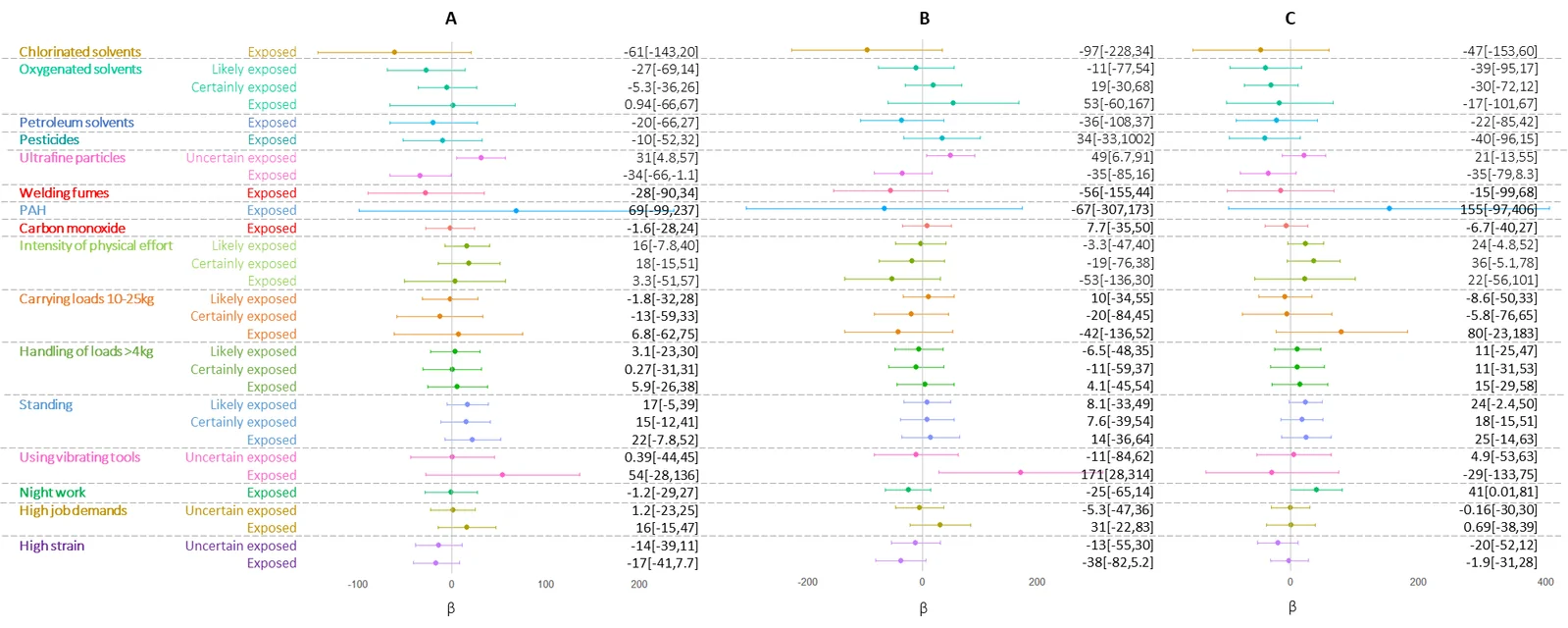
Relationship between occupational exposures and BW, for all women (A), women who stopped working during the first/second trimester of pregnancy (B), or the third trimester of pregnancy (C). Results of linear regression model adjusted for education level, tobacco, alcohol, BMI, infant’s sex and gestational age, with modality “not exposed” as the reference. Elfe study, France, N=12 579.

For all women, we observed a decrease in fetal BW among women exposed to UFP (β -34g, 95% CI -66– -1.1), high strain (β = -17g, 95% CI -41–7.7) and an increase in BW (β 31g, 95% CI 4.8–57) among women who were uncertain whether they had been exposed to them (figure 2A). Analysis by trimester of pregnancy showed similar results in terms of the direction of the association (figure 2B and 2C).

Among those who stopped working during the first/second trimester of pregnancy, we also observed that exposure to vibration was associated with an increase in BW (β 171g, 95% CI 28–314) (figure 2B).

Among those who stopped working during the third trimester of pregnancy, exposure to night work increased BW (β 41g, 95% CI 0.01–81) (figure 2C). We found that 23.1% of the women exposed to UFP were other service workers and 22.8% were technical saleswomen or commercial travelers. Of those exposed to vibrations, 27.4% were cleaners and helpers and 23.5% were hairdressers, beauticians. Of the participants, 31.3% of women were nurses, midwives, medical x ray technicians (supplementary appendix 7).

*HC.* Results of EWAS for HC are presented in supplementart appendix 8, table S3. Briefly, it revealed that exposure to chlorinated and oxygenated solvents was significantly associated with HC.

**Figure 3:**
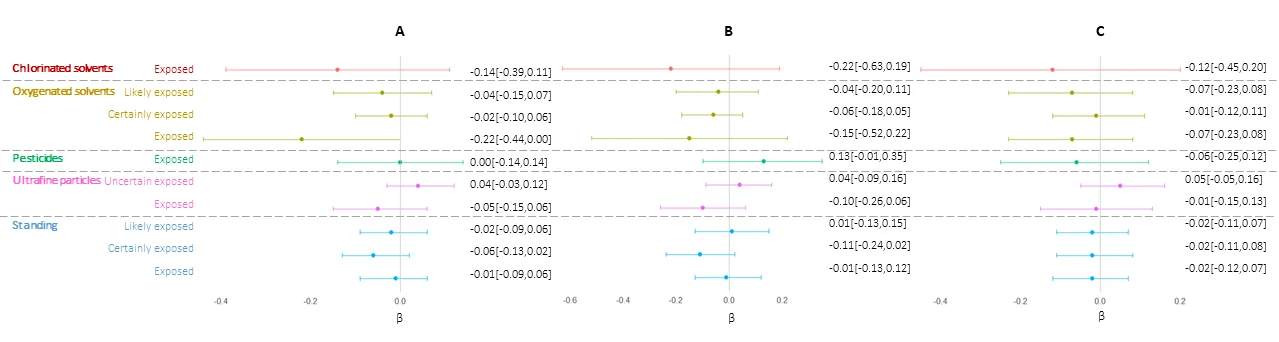
Relationship between occupational exposures and HC, for all women (A), women who stopped working during the first/second trimester of pregnancy (B), or the third trimester of pregnancy (C). Results of linear regression model adjusted for education level, tobacco, alcohol, BMI, infant’s sex and gestational age, with modality “not exposed” as the reference. Elfe study, France, N=11 412.

For all women, the model showed a decrease in HC (β -0.22cm, 95% CI -0.44–0.00) among the women exposed to oxygenated solvents (figure 3A). Analysis by trimester of pregnancy showed similar results in terms of the direction of the association (figure 3B and figure 3C).

Among those who stopped working during the first/second trimester of pregnancy, certainly exposure to standing decreased HC but this result was not significant (β -0.11cm, 95% CI -0.24–0.02) (figure 3B).

Among those who stopped working during the third trimester of pregnancy, no significant association was observed (figure 3C).

We found that 48.1% of the women exposed to oxygenated solvents were chemists, biologists, and related workers, and 32.1% were hairdressers, beauticians (supplementary appendix 7).

## Discussion

We identified some significant associations between occupational exposures and birth outcomes. Exposure to UFP was associated with increased risk of SGA and decreased BW, exposure to oxygenated solvents with decreased HC, and exposure to standing with decreased risk of SGA for all women included in the ELFE study. Among those who stopped working during the first or second trimester of pregnancy, exposure to vibrating tools was associated with increased BW and exposure to UFP and high strain with increased risk of SGA. Among those who stopped working during the third trimester of pregnancy, exposure to oxygenated solvents increased the risk of SGA, and exposure to night work was associated with increased BW. To the best of our knowledge, this is the first study to assess the contributions of a wide range of occupational exposures during pregnancy to intrauterine growth, based on hypothesis-driven variable selection.

We observed an association between exposure to UFP and decreased BW, increased risk of SGA for all women and those who stopped working during the first or second trimester of pregnancy. This result supported the findings of previous publications on the same population that revealed an increased risk of SGA (9, 10). Furthermore, a review by Yuan et al in 2019 (48) showed a link between exposure to PM2.5 and BW, SGA. Similarly, a meta-analysis on air pollution related to road traffic showed an association with SGA (49). This result is also in accordance with animal studies that reported the impact of UFP on the alveolo capillary barrier, bloodstream, and placental barrier which can be implicated in the occurrence of fetal growth restriction (50–55). We observed, in addition, that maternal occupational exposure to oxygenated solvents decreased HC among all women and increased the risk of SGA among women who continued working during the third trimester of pregnancy. Maternal occupational exposure to solvents has been associated in several studies with fetal growth retardation (2, 3). Using the same population study, a previous study reported these two associations using a single exposure approach, and both of these are confirmed by our study (2). The biological plausibility of this association is reinforced by animal studies that show fetotoxic and embryotoxic effects of several solvents. Experimental evidence indicates that some oxygenated solvents, particularly ethanol, can disrupt iron and zinc concentrations, which may reduce insulin-like growth factor 1 (IGF-1) levels and receptor activity. Given the key role of IGF-1 in fetal growth regulation, such alterations may contribute to intrauterine growth restriction (56). In addition, the effect of oxygenated solvents on decreased HC is also in accordance with our understanding of the neurotoxic properties of several solvents, which may interfere with brain development during critical period of gestation. We also found that occupational exposure to high strain increased the risk of SGA among women who stopped working during first/second trimester of pregnancy. This result is reinforced by experimental studies showing that the release of stress hormone like norepinephrine or cortisol impairs fetal growth dur to dysregulation of the hypothalamic-pituitary-adrenal axis (57, 58).

Our study revealed some significant associations with birth outcomes that were not as expected, specifically for night work associated with a lower risk of SGA and increased BW, as well as for vibration associated with increased BW. One possible explanation for these results may be the presence of a healthy worker effect. For example, women who continued night work later into pregnancy may represent a selected group with a better health status or more favorable pregnancy conditions, potentially contributing to better birth outcomes. A healthy worker effect in relation to pregnancy outcomes has already been reported (1).

We also note that, surprisingly, we did not observe any association in our model with postural constraints. Nevertheless, using the same population study in a previous study, we identified four profiles of maternal multi-exposures during pregnancy, and one, defined by postural constraints, psychosocial factors, was associated with birth outcomes (41). Several variables that were more widely represented in this profile were also identified with our a priori hypothesis and consequently included in our model: intensity of physical effort, standing, high job demands, and high strain. Mothers of this profile were also exposed to repetition of tasks and leaning forward or sideways, neither of which was included in the present study. Given that the profile mentioned above was associated with SGA and HC, it would be relevant to consider these two variables not included in our a priori analysis for future study.

In addition, we must acknowledge that we found no significant association for the other occupational exposures, whereas theses exposures were selected a priori based on epidemiological and/or experimental hypotheses. This can be explained by several reasons, including exposure measurement errors assumed to be non-differential, a low prevalence of some exposures, or low intensity of exposure in our cohort that will lead to reduced statistical power. In addition, we could not exclude that considering multiple exposures together may result in possible interactions with synergic or even antagonist effects between certain exposures. Nevertheless, when we conducted EWAS in this population study, only certain factors among those selected were associated with intrauterine growth: UFP for SGA, chlorinated solvents, intensity of physical effort for BW, and chlorinated and oxygenated solvents for HC.

We only selected one interaction (between carrying loads and high strain) based on the literature. This shows the lack of studies on this topic in the epidemiological literature and the potential need to investigate additional interactions by conducting a targeted review of the literature to identify the biological pathways involved. If several exposures share common mechanisms of action (for example, oxidative stress, placental inflammation, altered uterine perfusion), this would strengthen the plausibility of a cumulative or interactive effect.

Occupational exposure during pregnancy was assessed using JEM, which are standardized methods for exposure estimation. While JEM offer consistency across large populations, a key limitation is that they assign the same exposure level to all individuals within a given job category, without accounting for individual variability (59). The JEM used in our study have been constructed by experts (CANJEM, MatPUF, Pestipop, endocrine disruptor, Matgéné solvents, Mat-O-Covid, FinJEM, Eficatt), self-administered questionnaires (Matgéné night work, JEM Constances, Swedish psychosocial JEM), or literature-based measurements data combined with questionnaire data (RF-JEM). Although the declaration of subjects can be judged as less reliable than expertises, we have chosen to limit the use of JEM based on questionnaires to those targeting strenuous and psychosocial factors for which the declaration of subjects can be considered more valid than for chemical exposures. Some of the JEM used in our study, such as FinJEM and the Swedish psychosocial JEM, were developed using data from countries other than France, in which this study took place. This may have introduced some exposure misclassification. However, this potential bias is likely limited in the context of high-income countries with comparable occupational environments. Among the JEM applied, only the Swedish psychosocial JEM and night work JEM incorporated gender-specific estimates. For biomechanical exposures, the JEM Constances has shown comparable predictive performance between gender-specific and non-gender-specific models (60). Nonetheless, since many non-gendered JEM are traditionally based on male populations, some degree of exposure misclassification is possible. Several JEM include a temporal dimension to account for the evolution of work practices and regulatory changes, particularly regarding chemical exposures. By contrast, psychosocial and strenuous exposures were assumed to be temporally stable. The variability in JEM accuracy, along with the limited availability of robust tools for psychosocial and physical strain exposures, may have introduced non-differential measurement errors, which could have attenuated the strength of observed associations.

The generalizability of our results is limited to women who work during pregnancy, as we excluded women who did not work from the analysis. We chose to exclude these women to limit the healthy worker effect, as non-working women may have poorer health and a higher risk of adverse pregnancy outcomes (1).

This study has several strengths. First, it benefits from a large sample of participants from the ELFE cohort, with detailed data on women’s occupations during pregnancy, including work duration, relevant outcomes, and potential confounding factors. Second, the study provides detailed task descriptions, allowing a single expert to classify occupations according to international (ISCO-1968) and national (PCS-1994) occupational nomenclatures, as well as industrial activity classifications (CITI-1975 and NAF-2000). This approach enabled the use of multiple JEM to characterize and analyze a wide range of occupational exposures in our population study (41). A third strength was the a priori approach used to select occupational exposures related to fetal growth. Unlike data-driven methods, which can yield spurious associations due to multiple testing or overfitting, an a priori approach is grounded in existing scientific literature and biological plausibility. This method enhances the interpretability and relevance of findings by focusing on exposures previously hypothesized or demonstrated to affect fetal development. Nevertheless, we must acknowledge that only those exposures sufficiently investigated were eligible to be analyzed in our study. For instance long working hours is related to many occupational exposures, as well as behavioral factors like smoking (61, 62).

At the same time, this study has several limitations. The inclusion criteria for the ELFE cohort include live births beyond 33 weeks of gestation, thus excluding newborns with severe conditions such as prematurity. This selection therefore does not capture the most serious health effects, which may lead to underestimating certain effects or missing associations in the most severe cases. Although this is a large study compared to the international literature, we acknowledge that this sample did not allow for a sufficiently powerful study of infrequent occupational exposure. As discussed above, the use of JEM that may have introduced measurement errors. Moreover, statistical models for studying the occupational exposome remain quite limited today, particularly when considering categorical exposure variables. Indeed, other methods may be useful in this regard (42, 63), such as weighted quantile sum regression, quantile G-computation, or Bayesian kernel machine regression, all of which aim to evaluate the overall effect and interactions of a mixture of exposures. These methods are, however, only applicable to continuous exposure variables. Specific methods for studying qualitative exposure variables should be developed (42, 63–65). Finally, a large number of statistical tests were performed. This multiplicity of testing may have increased the risk of incorrectly identifying a statistical difference.

### Concluding remarks

This study highlights the potential effects of certain occupational exposures on birth outcomes (SGA, BW and HC), depending on which trimester of pregnancy women stopped working. By prioritizing exposures with known or suspected links to intrauterine growth, our a priori approach provides stronger grounds for public health recommendations. Although all the factors examined in this study have been associated with fetal growth in previous studies that have assessed them individually, their combination does not necessarily reveal any association. It would therefore be worthwhile to explore potential interactions between these factors by examining toxicological studies. Doing so would likely enable to better understand the pathways involved for each exposure.
